# Microwave Photon Detectors Based on Semiconducting Double Quantum Dots

**DOI:** 10.3390/s20144010

**Published:** 2020-07-19

**Authors:** Alberto Ghirri, Samuele Cornia, Marco Affronte

**Affiliations:** 1 Istituto Nanoscienze-CNR, via Campi 213/a, 41125 Modena, Italy; samuele.cornia@unimore.it (S.C.); marco.affronte@unimore.it (M.A.); 2 Dipartimento di Scienze Fisiche, Informatiche e Matematiche, Università di Modena e Reggio Emilia, via Campi 213/a, 41125 Modena, Italy

**Keywords:** microwaves, photon detectors, quantum dot

## Abstract

Detectors of microwave photons find applications in different fields ranging from security to cosmology. Due to the intrinsic difficulties related to the detection of vanishingly small energy quanta ℏω, significant portions of the microwave electromagnetic spectrum are still uncovered by suitable techniques. No prevailing technology has clearly emerged yet, although different solutions have been tested in different contexts. Here, we focus on semiconductor quantum dots, which feature wide tunability by external gate voltages and scalability for large architectures. We discuss possible pathways for the development of microwave photon detectors based on photon-assisted tunneling in semiconducting double quantum dot circuits. In particular, we consider implementations based on either broadband transmission lines or resonant cavities, and we discuss how developments in charge sensing techniques and hybrid architectures may be beneficial for the development of efficient photon detectors in the microwave range.

## 1. Introduction

Single-photon detectors find potential applications in several areas of physics and constitute relevant tools in the context of quantum technologies. For instance, in circuit Quantum Electrodynamics (cQED), microwave photon detectors may allow the remote entanglement of distant qubits or the development of quantum computation with photonic qubits [1]. Microwave photon detectors find application also in the search of dark matter particles for which the development of suitable detection techniques in the 5 to 500 GHz range is strongly demanded [2,3,4,5].

While in the visible range single-photon detection techniques are relatively well established [6], microwave photon counters have been reported only recently [7,8,9,10,11,12,13,14]. As a matter of fact, the detection of microwave photons is extremely challenging due to their small energy. Considering frequencies in the 1GHz<ω/2π<300GHz range, which in vacuum correspond to wavelengths 300mm>λ>1mm, the equivalent energy results 4μeV<ℏω<1.2meV. Such small values require low temperatures ($k_{B}T\ll \hbar \omega$) to suppress the thermal background. Just to fix some numbers: the energy of a photon at 10 GHz corresponds to a temperature of 480 mK, while at 300 GHz to 14.4 K.

Here, we draw our attention to the use of semiconductor quantum dots as microwave photon detectors. These can be easily integrated in electronic circuits and scalable architectures as required for multipixel detection. They have been widely investigated in the last decades for different purposes: several materials and device configurations have been tested and they show genuine quantum properties. The high tunability of simple Double Quantum Dot (DQD) structures by external gate potentials allows continuous tuning of energy levels spacings from GHz to THz frequencies. This is a remarkable feature that makes these systems complementary with respect to other quantum devices such as superconducting qubits. In DQDs, the absorption of a single photon drives electron transitions between the dots’ levels, resulting in net variations of the DQD conductivity that can be measured with high sensitivity and large bandwidth by means of suitable charge sensors. On the other hand, DQDs are prone to coupling to phonons and charge noise, thus relaxation and coherence times are shorter than those of superconducting qubits. An additional characteristic of DQD devices is their large electric dipole moment, which leads to large coupling strengths with the electric field component of a microwave resonator. Thanks to this feature, DQDs can be efficiently embedded in circuit quantum electrodynamics (cQED) architectures enabling fast manipulation and readout of charge or spin states by microwave fields. Based on these recent achievements, we discuss possible pathways to overcome limits and define next steps for the implementation of DQD-based microwave photon detectors.

## 2. State of the Art

**Figure 1:**
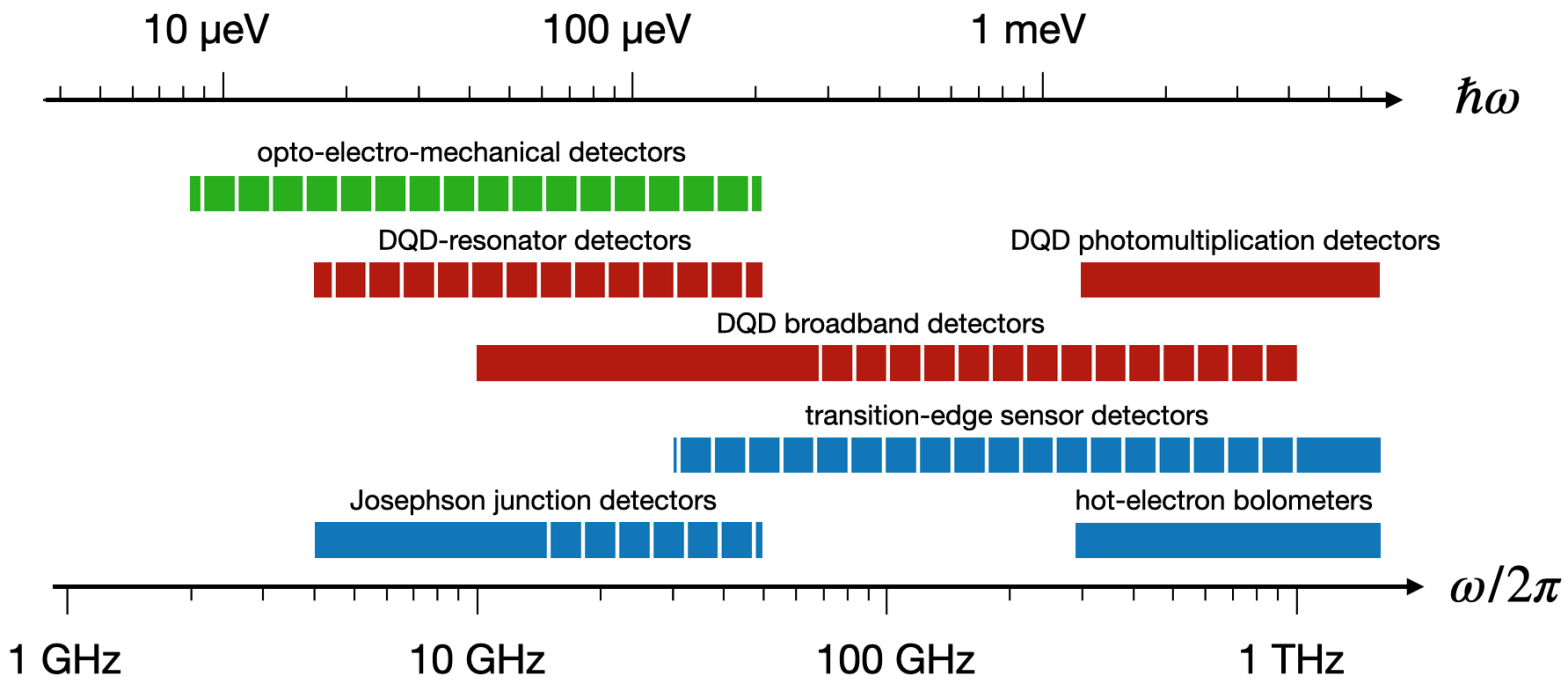
Schematic diagram showing the frequency working range of different photon detectors based on quantum dots (red), superconducting circuits (cyan), and opto-electro-mechanical systems (green). Solid regions show the frequency range of experimentally tested detectors, whereas striped regions indicate possible developments and theoretical proposals.

The working principle of photon detectors is based on the conversion of the impinging electromagnetic radiation into an electrical signal. Superconducting devices certainly represent a mature platform for the development of different types of photon detectors. Their working range in frequency is summarized in Figure 1. Superconducting qubits detectors have been successfully employed in the 4 to 20 GHz frequency range [1,7,8,9,10,11,12,13,14,15,16,17,18,19,20,21,22,23,24], which is in part related to the typical level spacing in superconducting quantum devices based on Josephson junctions [25], but also to the range in which control electronics is readily available. Future implementations will probably allow the extension of the maximum frequency up to ~50 GHz [26]. Worth mentioning is the development of quantum non-demolition detectors for either cavity [8,27] or itinerant [13,14] photons, which require no absorption and preserve the photon number [1]. Superconducting transition-edge sensors operating as bolometers show sharp resistance increase upon the absorption of photons [28]. These devices typically work at frequencies above THz, but extensions at frequencies down to 90 GHz are in progress [26]. Additionally, superconducting hot electron bolometers are operated above 300 GHz [28]. Detection schemes based on opto-electro-mechanical systems using mechanical resonators with coupled microwave and optical cavities have also been theoretically proposed [29,30,31].

Photon detectors based on semiconductor quantum dots (QDs) have been proposed and realized for a wide range of frequencies (Figure 1). Single-photon detectors based on photomultiplication work at frequencies above 300 GHz (Section 3). Detectors based on photon-assisted tunneling have been reported for lower frequencies. Noise detectors were developed to investigate quantum noise excitations in quantum point contacts and they work in the 10 to 80 GHz frequency range. Given the single particle energy spacing in specific DQD devices, this upper bound can be significantly extended up to THz (Section 4). In order to increase the sensitivity, it has been proposed to couple the DQD with a high-quality factor resonator (Section 5). In this case, the frequency range of the detector is determined by the characteristics of the resonator. Superconducting coplanar resonators are usually fabricated with fundamental frequency ranging between ≈100 MHz and 20 GHz. By employing shorter resonators, higher-order harmonics, or three-dimensional waveguide cavities, an extension of the maximum working frequency up to ~50 GHz can be envisaged.

## 3. Detection of Sub-Millimeter Wave Photons by DQDs

**Figure 2:**
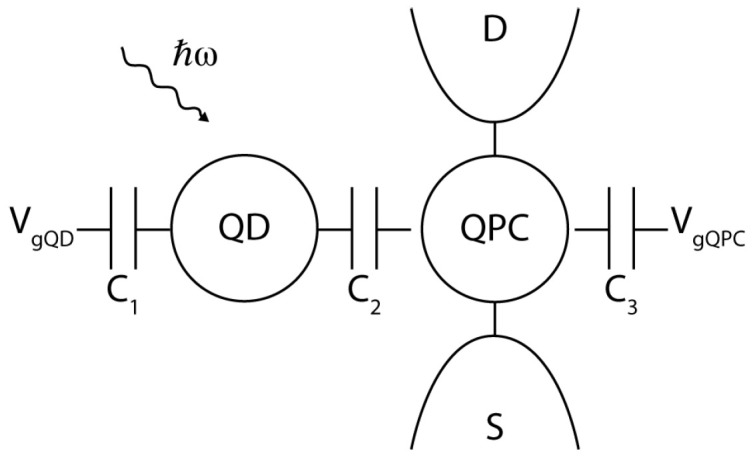
Capacitively coupled systems made by two quantum dots (QDs)—or a QD and a quantum point contact (QPC)—are used to detect photon absorption events. In this scheme, the two systems are individually tuned by external gates. The left dot works as the absorber, the second device is tuned in a configuration where its conductance is strongly dependent on the electrostatic environment. The transition induced by the absorption of the photon results in a conductance change for the sensing (QPC) device.

Semiconductor QDs have been tested as photon detectors at sub-millimeter wavelengths [32]. The first demonstration of single photon sensitivity has been achieved in the 300 to 600 GHz range by exploiting the transitions between Landau levels in GaAs/AlGaAs QDs in the presence of a high magnetic field [33]. Further experiments focused on devices in which a first dot is coupled to a planar sub-millimeter wave antenna and the second dot, capacitively coupled to the first, is electrically connected to external leads in order to work as single-electron transistor (SET) [34] (see Figure 2). The device essentially works as a photomultiplier. The absorption of photons at frequency ~500 GHz by the first dot determines telegraph-like switches of the conductance peaks of the DQD, which are acquired by dc conductivity measurements with timing resolution in the millisecond range, thus much lower than the response time of the DQD. The current responsivity of such a photon detector, being defined as the ratio between the measured current signal and the incident microwave power, results as $R=eG_{PC}\eta /\hbar \omega$, where $G_{PC}$ is the photoconductive gain, i.e., the number of photoelectrons generated by each impinging photon, and η<1 is the quantum efficiency [32]. Values of $G_{PC}$ in the $10^{5}$ to $10^{12}$ range have been reported for frequencies above 500 GHz, giving rise to values of *R* similar to what is obtained by conventional phototubes in optics [32]. To our knowledge, photomultiplication effects have not been reported below 300 GHz as in this range $G_{PC}$ has typically unit value.

## 4. DQD Broadband Microwave Photon Detectors

### 4.1. DQD Noise Detectors

**Figure 3:**
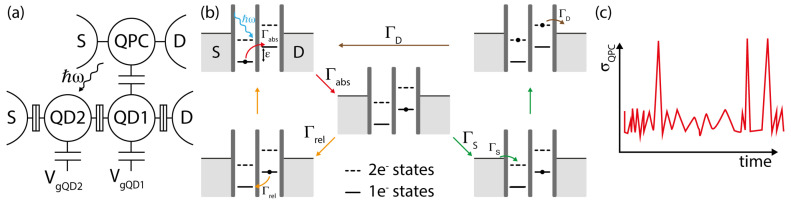
(**a**) Noise detection scheme based on quantum dots (QDs). The quantum point contact (QPC), which is capacitively coupled to the double quantum dot, acts both as a source of microwave photons and as a charge sensor that probes the configuration of the DQD. (**b**) Scheme of the different paths that the DQD system can take after photon absorption. $\Gamma_{rel}$ shows the relaxation path, where the electron returns to the ground state, emitting a phonon/photon in the process. $\Gamma_{S}$ followed by $\Gamma_{D}$ shows the charging with an additional electron through tunneling from the source contact. In the two-electron state, tunneling out of the device is permitted: this returns the system to the initial configuration. (**c**) Typical time trace of the detector signal. The peaks correspond to entering and leaving of the additional electron in the DQD.

The absorption of microwave photons in a QD gives rise to inelastic electron transitions that occur either between the electronic reservoirs in the leads and the energy levels in the single dot, or between the DQD’s discrete energy levels themselves. In general, these processes are reported as photon-assisted tunneling (PAT) [35,36,37,38]. PAT in QDs can be used to develop frequency-selective detectors of quantum noise, as demonstrated by means of quantum point contact (QPC) charge detectors [39,40,41,42,43]. These experiments showed the possibility to measure the photon absorption rate in a frequency range between 10 and 80 GHz with time-resolved measurements of the DQD conductivity [42,43]. The working principle is sketched in Figure 3. The left and right dot levels are detuned in such a way that their energy separation (ε) matches the frequency of the incoming photons, while source-drain bias voltage ($V_{SD}$) is set to zero. When the QPC is polarized, it emits microwave photons by shot noise that are subsequently absorbed by the DQD, giving rise to PAT transitions from the left to the right dot with characteristic rate $\Gamma_{abs}$. The electron then relaxes back to the initial state with typical relaxation rate $\Gamma_{rel}=1/T_{1}∼60$ MHz. These “internal” transitions are too fast to be directly detected by the nearby QPCs (bandwidth 30 kHz), thus they are not visible in the measured time trace. However, as lead-dot tunnel coupling can be $\Gamma_{l}=1$ kHz $\ll \Gamma_{rel}$ as in [42], an additional electron can occasionally enter and leave the DQD at a slower rate. In this case, such “external” transitions can be effectively detected by the QPC and the measured rates can be related to the internal DQD transitions, allowing the estimation of the photon absorption rate $\Gamma_{abs}$[42,43]. With this protocol, the detector efficiency is proportional to the $\Gamma_{l}/\Gamma_{rel}$ ratio and amounts to $\eta ∼10^{-5}$ [43]. Fast detection circuits and improved DQD relaxation times are thus required in order to improve the efficiency of the detector.

### 4.2. Charge Sensing of DQDs

**Figure 4:**
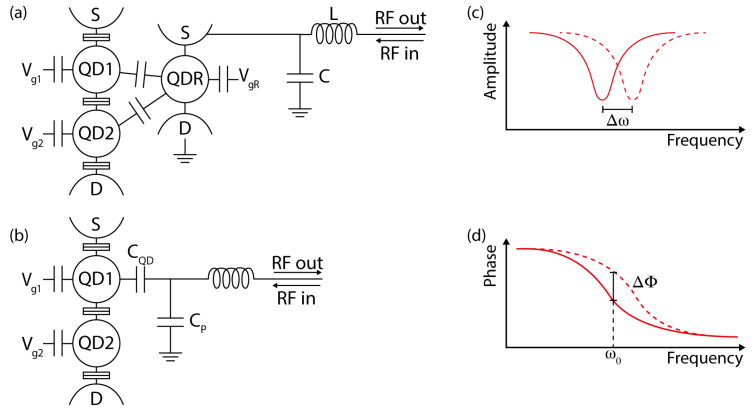
Schematics of two different reflectometry techniques. Panel (**a**) shows the rf-SET setup. A read-out quantum dot (QDR) is capacitively coupled to the double quantum dot, with different couplings to each dot. The single-electron transistor (SET) is highly sensitive to the charge configuration of the DQD thanks to the sharpness of the tunneling resonances. Configuration changes due to DQD tunneling events result in a strong change in the SET conductance. This can be observed by monitoring the signal reflected by the resonating circuit. Panel (**b**) shows the gate reflectometry configuration. In this case, the resonating circuit is directly connected to one of the gates controlling the DQD. Tunnel coupling between the QDs determine a change of the quantum capacitance that can be resolved as a frequency shift of the resonating circuit. This approach results in a simplified and more compact device, but is less performant as a readout technique. In both approaches a bias tee can be used to dc bias the rf lines, so that they can provide a source-drain bias or configure the QD (substituting $V_{gQD1}$ in this case). Panels (**c**,**d**) show the expected change in the reflected signal amplitude and phase in different configurations (dashed and non-dashed lines) for these readout schemes.

A fundamental aspect for the development of semiconductor detectors is the realization of fast charge sensors. A QPC or a SET capacitively coupled to the DQD can provide highly sensitive measurements of the source-drain conductivity [38,44,45,46]. However, the measurement bandwidth of these devices typically has a high-frequency cut-off of less than 100 kHz due to the RC time constant of the cryogenic wiring and to the limited bandwidth of the current-to-voltage converter. To overcome these limitations, radio-frequency (rf) SET reflectometry was developed [47,48,49,50,51,52]. Fast and high-fidelity readout of the DQD is obtained by incorporating the charge sensor into an impedance matching tank circuit: changes to the electrostatic potential of the charge sensor alter its conductance and therefore generate measurable changes to the reflection coefficient of the circuit (Figure 4). As an example, charge transitions in few-electrons GaAs DQDs were resolved in single-shot measurements with an integration time of 100 ns and signal-to-noise ratio equal to 3 [53]. Rf reflectometry has been applied also on InAs nanowire [54,55,56] and Si/SiGe DQDs [57,58].

As a possible alternative, gate reflectometry bridges the gap between cQED and rf reflectometry [59,60,61,62]. It makes use of lumped element sub-GHz resonators to probe changes in the tunneling capacitance due to device configuration. These resonators are typically formed by an off-chip inductance (*L*) and a total capacitance (*C*) that is the result of parasitic and QD contributions. The dot tunneling-dependent quantum capacitance [63] leads to a shift in the resonance frequency ($\omega_{0}=1/\sqrt{LC}$). The resonator is usually probed in the dispersive regime, where the reflected signal experiences a phase shift, consistently with a cQED input–output approach. While being currently less performant than rf-SET in terms of fidelities and bandwidth [64], this technique has the advantage of requiring a simpler device design, as the resonator is connected directly to one of the gates defining the QD (with the highest lever arm possible in order to maximize the shift) instead of requiring an additional SET/QPC nearby.

Full cQED approaches employing a superconducting resonator have been used, in a similar fashion to what is done with superconducting qubits, to perform dispersive readout of charge and spin states. In the dispersive regime, the phase response of the resonator is sensitive to the DQD configuration. When the DQD is far from transitions between (M,N) charge states, its characteristic energy is typically orders of magnitude higher than the resonator frequencies and the two are far detuned. When the DQD is close to an interdot charge transition with energy slightly detuned from the cavity frequency, the dispersive interaction leads to a state-dependent frequency and phase shift. The same is true for spin states in presence of a magnetic field, when the resonator is detuned from a spin transition. More quantitatively, we consider a transition between DQD levels with frequency splitting $\omega_{\sigma }$. When the DQD-resonator detuning $\Delta \omega =\omega_{\sigma }-\omega_{0}$ is $\Delta \omega >g_{c(s)}$, where $g_{c(s)}$ is the photon-charge (photon-spin) coupling strength, the phase shift at bare cavity frequency is $\Delta \phi =-\arctan\left(2g_{c(s)}^{2}/\kappa \;\Delta \omega \right)$, where κ is the cavity decay rate. This approach can be used to map the DQD stability diagram even if no bias is applied [65,66], and therefore the leads do not need to be connected to an electron reservoir, similarly to gate-reflectometry. This technique was successfully used to perform qubit state read-out for charge qubits [67], spin qubits [68], singlet-triplet qubits [65], and exchange qubits [69].

### 4.3. Detection of Microwave Photons by Conductivity Measurements

The noise detectors described above can potentially be implemented also for the detection of itinerant microwave photons. For this purpose, DQDs are coupled to a transmission line providing the flux of impinging photons. In microwave spectroscopy of DQDs [37], this has been carried out by introducing a capacitor between one of the gate electrodes and the coaxial line. This approach can be considered widely tunable as the coaxial line allows broadband transmission of photons, whereas the DQD can be tuned in the selected detection window. For these applications, DQD devices with large single-particle excitation energies, such as those implemented in InAs nanowires [70,71,72,73], appear particularly interesting for the possibility they offer to continuously tune the DQD levels from few GHz to THz frequencies.

Time-resolved detection of single PAT transitions requires sensitive and fast detection. QPCs would play this role but they are themselves source of microwave photons [40,42]; in a photon detector this would increase dark counts unless the detection frequency is set beyond the cut-off frequency of the QPC noise generator [40]. Moreover, QPCs typically show a response much slower than the relaxation rate of DQDs. In this respect, rf reflectometry would perform better, given that charge sensing with bandwidth up to ~1.5 MHz [53] has been reported for GaAs and gate sensing with 1 μs integration time has been reported for InAs DQDs [62]. The reflectometry technique has been recently implemented also for Si/SiGe DQDs, demonstrating single-shot singlet-triplet readout with an integration time of 0.8 μs [58]. For Si/SiGe DQDs, the charge relaxation time was shown to vary over four orders of magnitude as a function of detuning and interdot tunneling parameters, with a maximum value $T_{1}=45$ μs [74]. These results indicate that rf reflectometry can be implemented to sense the DQD at rates faster than the charge relaxation times, thus opening a way to the realization of efficient DQD based microwave photon detectors.

Coupling with acoustic phonons is strong in DQDs and represents the primary source of dark counts of the detector [75]. This effect is more pronounced in nanostructured DQDs, where strong electron–phonon coupling follows as a consequence of tight electronic confinement and characteristic phonon environment of the nanostructures [76,77].

Due to the small size of dots (10–500 nm) compared to typical wavelength of MW photons (1–300 mm), their quantum yield, i.e., the efficiency to transduce MW photons to electrical signal, is generally low for bare QDs. To improve this figure of merit, coupling with resonators is a valid solution as we discuss in the following.

## 5. Photon Detectors Based on DQDs Coupled to a Microwave Cavity

### 5.1. Coupling of DQD to a Single Mode Resonator

**Figure 5:**
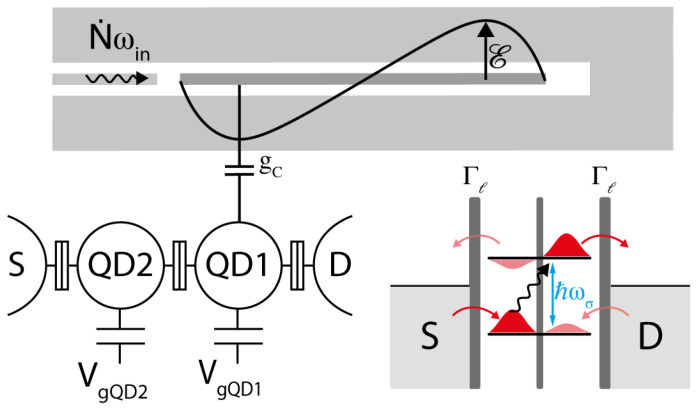
Schematics of the hybrid DQD-resonator circuit. A transmission line with incident photon flux $\dot{N}\omega_{in}$ is capacitively coupled to a high-quality factor coplanar waveguide resonator that behaves like an ideal quantum harmonic oscillator, with low average photon occupation. The resonator is also capacitively coupled ($g_{c}$) to the DQD. The level spacing of the DQD can be tuned to match the resonator frequency. When a photon is absorbed, the excited electron can tunnel to the drain contact and be detected as a current flow.

cQED architectures with DQD devices embedded in a high-quality factor resonator have been investigated for the coherent manipulation of DQD charge and spin states [78]. Superconducting coplanar waveguide resonators have demonstrated high versatility for coupling two-level quantum systems to confined microwave fields [79]. They show fundamental frequency ($\omega_{0}$) in the GHz range and internal quality factor ($Q_{int}$) reaching values above $10^{6}$ for bare resonators fabricated with optimized procedures [80,81]. The capacitive coupling to external transmission lines (Figure 5) gives rise to the external quality factor $Q_{ext}$. The loaded quality factor is given by $1/Q_{L}=1/Q_{int}+1/Q_{ext}$ and the photon decay rate is $\kappa =\omega_{0}/2\pi Q_{L}$ [82].

In a DQD, in the case of weak interdot tunnel coupling ($t_{c}$), the electrons are strongly localized on the individual left dot (|L〉) and right dot (|R〉) states. Conversely, for larger $t_{c}$, the DQD can be described as a two-level system, whose eigenstates [37]

(1) $$\begin{array}{cc} |g\rangle = & \cos\frac{\theta }{2}|L\rangle -\sin\frac{\theta }{2}|R\rangle \end{array}$$

(2) $$\begin{array}{cc} |e\rangle = & \sin\frac{\theta }{2}|L\rangle +\sin\frac{\theta }{2}|R\rangle , \end{array}$$

are often referred as bonding and antibonding states. Their energy spacing is

(3) $$\hbar \omega_{\sigma }=\sqrt{4t_{c}^{2}+\varepsilon^{2}},$$

where ε is the detuning energy, equal to the difference in the chemical potentials of the two dots, and $\cos\theta =-\varepsilon /\hbar \omega_{\sigma }$. The interaction between DQD and superconducting resonator can be modelled by a Jaynes–Cummings Hamiltonian, which, in the rotating-wave approximation and for $\omega_{\sigma }\approx \omega_{0}$, reduces to [83]

(4) $$H=\frac{\hbar \omega_{\sigma }}{2}\sigma_{z}+\hbar \omega_{0}a^{†}a+\hbar g_{0}\sin\theta \left(\sigma^{†}a+a^{†}\sigma \right)$$

where σ=|g〉〈e| is the DQD lowering operator, and $\sigma_{z}=\left|e\rangle \langle e\right|-\left|g\rangle \langle g\right|$. *a* and $a^{†}$ are the photon annihilation and creation operator, respectively. The capacitive electron–photon coupling depends on the configuration of the dot in terms of $t_{c}$ and ε, and results $g_{c}=g_{0}\sin\theta =2g_{0}t_{c}/\sqrt{4t_{c}^{2}+\varepsilon^{2}}$. $g_{0}$ is the coupling strength given by

(5) $$g_{0}=\omega_{0}v\sqrt{\frac{2Z_{0}}{R_{Q}}},$$

where $v=C_{c}/\left(C_{c}+C_{\Sigma }\right)$, $C_{c}$ being the gate capacitance between the resonator and DQD, and $C_{\Sigma }$ the total capacitance of the DQD; $Z_{0}=\sqrt{L/C}$ is the characteristic impedance of the resonator, which is related to the resonator’s inductance (*L*) and capacitance (*C*) per unit length; and $R_{Q}=h/e^{2}\approx 26$ kΩ is the resistance quantum.

The typical parameters obtained for different DQD devices are summarized in Table 1. Experimentally, the coupling between resonator and DQD is obtained by connecting the central conductor of the coplanar resonator to one of the DQD electrodes. Lumped-element low-pass filters can be introduced between the central conductor of the resonator and the dc voltage source in order to implement dc bias of the dot gate used for the coupling [84]. Values of DQD-resonator coupling ($g_{c}$) from a few MHz up to 200 MHz have been reported for different hybrid DQD-resonator devices. $g_{c}$ is typically maximum for ε=0 due to the strong electric dipole moment of the DQD at the charge degeneracy point. In order to maximize $g_{c}$, the resonator can be designed to achieve large zero-point electric field fluctuations ($E_{rms}$). Being $E_{rms}\propto \omega_{0}\sqrt{Z_{0}}$, different approaches have been reported to increase $Z_{0}$, including higher-impedance coplanar resonators [68], high-impedance resonators with SQUID arrays [85], or high-kinetic inductance NbTiN nanowire resonators [69,86,87]. The coupling of the spin degree of freedom requires presence of either spin orbit interaction [65,88,89] or an inhomogeneous magnetic field [68,90,91], thus the spin coupling strength ($g_{s}$) can be an alternative to bare electrical $g_{c}$. The regime of strong DQD–photon coupling has been achieved with either charge ($g_{c}\gg \gamma_{c},\kappa$) [67,85,92] or spin ($g_{s}\gg \gamma_{s},\kappa$) [68,69,86] qubits.

The damping rates $\gamma_{c}$ and $\gamma_{s}$ are related to relaxation and dephasing of the DQD charge and spin state, respectively. A large variation of these parameters is reported for different DQD devices and materials (Table 1). Fluctuating electric fields affects both relaxation and dephasing rates in DQD charge and spin qubits. Electric field fluctuations could arise from different sources, including background 1/f charge noise, fluctuations in the gate potentials or other electrical noise sources [97,98,99,100]. Dephasing in DQD charge qubits is more affected by charge noise for ε≠0. Conversely, for ε=0, the DQD energy is insensitive to gate potential fluctuations (“sweet spot”) at first-order [98]. Charge noise induced dephasing is proportional to the square of the total charging energy $E_{c}^{2}$, with $E_{c}=e^{2}/C_{\Sigma }$ [99], thus larger interdot capacitance, i.e., smaller interdot charging energy, is expected to reduce the effect of charge noise [67,101]. Coupling to the phonon bath can also induce fluctuating electric fields as an effect of different mechanisms. Common to all semiconductors is the inhomogeneous deformation of the crystal lattice under the effect of the so-called deformation potential phonons, which alter the band gap in space and give rise to fluctuating electric fields. Additionally, in polar crystals, such as III-V semiconductors, homogeneous strain leads to electric fields through the piezoelectric effect [97].

### 5.2. Photon Detection by DQD Coupled to a Microwave Resonator

A microwave photon detector based on a DQD coupled to a microwave cavity has been recently theoretically proposed [102]. The schematic diagram of the detector is shown in Figure 5. A flux of $\dot{N}$ microwave photons with frequency $\omega_{in}$ entering from a transmission line is stored in a single-port resonator with high-quality factor ($Q_{L}$) and frequency $\omega_{0}$. The DQD detector is appropriately configured near the charge transitions between the charge states |L〉=|N+1,M〉 and |R〉=|N,M+1〉, where |N,M〉 denotes N(M) electrons in the left (right) dot. In the “pumping” configuration, with $V_{SD}=0$ and nonzero detuning (ε≠0), inelastic PAT transitions lead to pumping electrons between the left and right dot, or vice versa, depending on the sign of ε, thus giving rise to a net source-drain current [37]. The spacing of the DQD levels is tuned to the frequency of the input photons ($\omega_{in}=\omega_{0}$) for $\varepsilon =\varepsilon_{res}=\sqrt{\omega_{in}^{2}-4t_{c}^{2}}$. Tunnel couplings are also appropriately tuned in order to ensure that the dot-lead tunneling is faster than the DQD relaxation rate.

For symmetric dot-lead tunneling with rate $\Gamma_{l}$, the photon-induced current $\langle \Delta I\rangle =\langle I-I_{0}\rangle$, where $\langle I_{0}\rangle$ is the current generated by dark counts for $\dot{N}=0$, reads [102]

(6) $$\langle \Delta I\rangle =-e\frac{\Gamma_{l}\cos\left(\theta \right)}{3}\Delta m_{z}.$$

Here, $\Delta m_{z}=m_{z}-m_{0}$ is the photon-induced polarization, being

(7) $$m_{0}=-\frac{\gamma_{e}+\Gamma_{l}}{3\gamma_{1}/2-\gamma_{e}/2+\Gamma_{l}}$$

the equilibrium polarization. $\gamma_{1}=1/T_{1}$ is the incoherent relaxation rate, where $T_{1}$ is the charge relaxation time, and $\gamma_{e}$ is the phonon-induced spontaneous emission rate due to difference in excitation and relaxation rates related to thermal phonons [102]. The effective depolarization rate results

(8) $$\Gamma_{1}=\gamma_{1}+\frac{2\Gamma_{l}-\gamma_{s}}{3},$$

while the transverse relaxation rate reads

(9) $$\Gamma_{2}=\frac{1}{2}\left(\gamma_{1}+\Gamma_{0e}\right)+\gamma_{\phi },$$

where $\Gamma_{0e}$ is the incoherent tunneling rate to the lead. The dephasing induced by low-frequency 1/f charge noise ($\gamma_{\phi }$) is also included to quantify its contribution to $\Gamma_{2}$.

By using the relaxation parameters of Si/SiGe DQDs ($T_{1}=10$ ns for $\omega_{\sigma }/2\pi =12$ GHz [74]), the calculated photon-induced current results $\Delta I=0.16\eta \dot{N}$ pA/MHz for $\dot{N}$ comprised between 1 MHz and 100 MHz [102]. The detection process takes advantage of fast dot-lead tunneling rate relative to DQD inelastic decay rate: for a flux $\dot{N}=1$ MHz the maximum efficiency is obtained for dot-lead tunneling rate $\Gamma_{l}=1$ GHz. This value is a trade-off between low $\Gamma_{l}$, for which loss of polarization due to the relaxation from |e〉 back to |g〉 is expected, and high $\Gamma_{l}$ which induces level broadening.

The detector efficiency, defined as $\eta =\left|\langle \Delta I\rangle \right|/e\dot{N}$, has been calculated by optimizing the reflection of input photons at the resonator port [102,103]. The reflection is minimized when the decay rate of the resonator (κ) matches the DQD-mediated photon dissipation rate

(10) $$\kappa =\frac{2{\left[g_{c}\left(\varepsilon_{res}\right)\right]}^{2}\left|m_{z}\right|}{\Gamma_{2}\left(\varepsilon_{res}\right)},$$

where $g_{c}\left(\varepsilon_{res}\right)=g_{0}\sin\theta_{res}=2g_{0}t_{c}/\sqrt{4t_{c}^{2}+\varepsilon_{res}^{2}}$. When this condition is valid, the detector efficiency becomes

(11) $$\eta_{res}=\left|\cos\theta_{res}\right|\frac{2\Gamma_{l}/3\Gamma_{1}}{1+2\dot{N}/\left|m_{0}\right|\Gamma_{1}}.$$

In the optimized condition, obtained by combining the parameters $t_{c}/h=0.5$ GHz, $g_{0}=50$ MHz, and κ/2π=76 kHz, the calculated efficiency is η≈98% even in the presence of strong DQD dissipation [102]. The depolarization rate that determines $\eta_{res}$ through Equation (11) is $\Gamma_{1}∼\Gamma_{l}$, so long as the charge relaxation rate is $\gamma_{1}\ll \Gamma_{l}$ (Equation (8)). The dephasing rate $\gamma_{\phi }$ does not directly affect the detector efficiency $\eta_{res}$, because the effect of a large $\gamma_{\phi }$ can, in principle, be compensated by a smaller κ (Equation (10)). In particular, the achievement of strong DQD–photon coupling is not required but high efficiency is obtained by combining state-of-the-art $g_{c}$ values (Table 1) with high $Q_{L}∼10^{5}$.

### 5.3. Experimental Realization of DQD-Resonator Microwave Photon Detectors

The detector proposed by Wong and Vavilov [102] and summarized in the previous section potentially allows high detection efficiency with realistic DQD and resonator parameters. The decay rate of the resonator (κ), thus the loaded quality factor $Q_{L}$, remains the most stringent parameter. Table 1 summarizes the typical values of $Q_{L}$ reported so far in different experiments. Largest $Q_{L}$ have been reported for Nb coplanar resonators and are typically comprised between 2000 and 10,000. SQUID array resonators and NbTiN high kinetic impedance resonators, although showing larger DQD-resonator coupling $g_{c}$, have so far displayed lower quality factors. In both cases, the reported $Q_{L}$ are lower than what has been reported for bare coplanar waveguide resonators [82]. This discrepancy is partially related to $Q_{ext}$, which can be controlled within a certain range in order to obtain the desired coupling to the external transmission lines. In any case, the introduction of DQD device and leads within the resonator mode volume causes a decrease of the internal quality factor. Strategies to improve $Q_{int}$ have focused on the introduction of low-pass LC filters between DQD gates and the dc voltage source in order to reduce the photon leakage through the dc bias lines of the DQD [84].

As mentioned above, the extension of the working range of the DQD-resonator detector to frequencies ω>10 GHz requires cavities with higher resonance frequency. Although coplanar waveguide resonators have typically been employed for frequencies below 20 GHz [104], higher fundamental frequencies can be obtained for short (length ≈2 mm or less) coplanar resonators [105]. Higher-order harmonics can be also potentially used. As an example, higher order modes of a Nb coplanar resonator with fundamental frequency at 5 GHz have been employed for spectroscopic studies up to 50 GHz [104]. High-frequency planar resonators with different structures were also proposed [106,107]. In general, the quadratic frequency increase of the surface resistance of the superconductor gives rise to a progressive reduction of the loaded quality factor with the increasing frequency [82].

Alternatively, the necessity to conjugate DQD devices with high-quality factor resonators may be addressed by developing hybrid architectures with three-dimensional cavities. Three-dimensional micromachined superconducting cavities have been realized for experiments with a transmon qubit at 8 GHz [108], showing a superior degree of environmental isolation and quality factors exceeding $10^{6}$ [109,110,111]. High-quality factor millimeter wave cavities based on the intersection of evanescent waveguides have been also recently developed for operation at frequency up to ~100 GHz [112]. First attempts to place a GaAs DQD in a waveguide cavity have already been reported [113], however showing weak DQD-cavity coupling and a decrease of the quality factor caused by the introduction of dc lines within the cavity mode. Different geometries, based on a split cavity design and on the introduction of the electrodes on nodes of the microwave electric field, have been investigated and shown to minimize the decrease of the internal quality factor [114,115]. Further experimental studies are necessary in order to optimize the DQD-cavity design and to improve both coupling and quality factor.

## 6. Conclusions and Outlook

Double quantum dot devices represent a fully configurable platform that can be implemented for the conversion of microwave photons into electric signals. Photons absorbed by the DQD give rise to photon-assisted tunneling transitions between the DQD levels and to measurable source-drain currents. On the basis of recent experimental and theoretical results, we have discussed possible solutions for the realization of microwave photon detectors based on DQD devices. We have considered both DQD detectors coupled to a broadband transmission line and DQD detectors coupled to a microwave resonator. The former derives from DQD noise detectors and potentially allows the detection of itinerant photons in a transmission line coupled to the DQD. The detector spectral range can be varied in a wide frequency interval thanks to the high tunability of the DQD energy level spacing. In order to achieve high detection efficiency, such broadband detectors require DQDs with long charge relaxation times along with the presence of fast and sensitive readout circuits. Conversely, photon detectors based on DQDs coupled to a microwave resonator are closely related to hybrid semiconductor–superconductor circuits developed in the context of cQED. Here, photons are stored in the resonator, whose features determine frequency and bandwidth of the photon detector. Optimized conditions require large DQD relaxation times, large DQD-resonator coupling strengths and low resonator decay rates. Under these conditions, the theoretically calculated detection efficiency exceeds 98%.

In both cases, the achievement of high detection efficiency is potentially feasible, but requires state-of-the-art DQD devices, fast charge sensing techniques and efficient coupling of the DQD to a high-quality factor resonator. These achievements stem from the advances in closely related research fields, ranging from semiconductor quantum devices to cQED architectures. DQD-based circuits are potentially useful in order to realize photon detectors working between 10 and 300 GHz, a range in which efficient detection techniques are presently missing. Experiments with real devices will be useful in order to evaluate efficiency and dark count rate and to compare these numbers with those obtained with other microwave photon detectors, in particular superconducting circuits.

## Figures and Tables

**Table 1:** Summary of the typical parameters reported for different DQD devices coupled to superconducting planar resonators.

DQD	Res.	$\frac{\omega_{0}}{2\pi }$ (GHz)	$Q_{L}$	$\frac{2t_{c}}{h}$ (GHz)	$\frac{g_{c}}{2\pi }$ (MHz)	$\frac{\gamma_{c}}{2\pi }$ (MHz)	$\frac{g_{s}}{2\pi }$ (MHz)	$\frac{\gamma_{s}}{2\pi }$ (MHz)	Ref.
GaAs	Al	6.755	2630	9	50	900	-	-	[66]
InAs NW	Nb	6.2	2000	1.8–7	30	5100	-	-	[65]
CNT	Al	6.72	3500	5.5	3.3	550	-	-	[93]
Graphene	Al	6.23896	3100	6.4	6	400	-	-	[94]
CNT	Nb	6.75	9650	-	-	-	1.3	2.5	[91]
GaAs	Al	6.852	2058	7.4	11	250	-	-	[95]
InSb NW	Nb	6.0749	8000	7	14	1000–4000	-	-	[96]
Si	Nb	7.684	7460	7.68	6.7	2.6	-	-	[92]
GaAs	SQUID	5.03	401	4.13	119	20	-	-	[85]
Si	Nb	5.846	4700	4.9, 7.4	40	35	5.3	2.4	[68]
Si	NbTiN	6.051	1120	12.6	200	52	13	2.5	[86]
GaAs	SQUID	5.07	169	3.3	57	3.3	-	-	[67]

## Abbreviations

The following abbreviations are used in this manuscript.

MW	Microwave
QD	Quantum dot
DQD	Double quantum dot
QPC	Quantum point contact
SET	Single electron transistor
cQED	Circuit quantum electrodynamics
dc	Direct current
rf	Radio frequency
NW	Nanowire
CNT	Carbon nanotube
Res	Resonator
